# Editorial for the Special Issue: “Characterization and Processing of Complex Materials”

**DOI:** 10.3390/ma16103830

**Published:** 2023-05-19

**Authors:** Akira Otsuki

**Affiliations:** 1 Facultad de Ingeniería y Ciencias, Universidad Adolfo Ibáñez, Diagonal Las Torres 2640, Peñalolén, Santiago 7941169, Chile; akira.otsuki@uai.cl; 2 Waste Science & Technology, Luleå University of Technology, SE 971 87 Luleå, Sweden; 3 Neutron Beam Technology Team, RIKEN Center for Advanced Photonics, RIKEN, Wako 351-0198, Japan

## 1. Introduction

The Special Issue aimed to provide a forum for scientists and engineers to share and discuss their pioneering/original findings or insightful reviews on the “Characterization and Processing of Complex Materials”. Reports on the achievement of coupling the complexity of industrial materials/wastes/natural ores with non-destructive characterization method(s) and the unique advancement of their processing were particularly encouraged. On the other hand, any kind of contribution under the broad framework of the understanding and production of complex materials was also highly regarded.

The Special Issue was intended to gather papers on the characterization and processing of complex materials with the following keywords: non-destructive characterization; analytical chemistry and instrumentation; waste management and recycling; selective material recovery/processing; sustainability and circular economy. One of them is the waste management and recycling of complex materials (e.g., foods and electronic wastes) via the development of a new characterization method and/or process intensification. For example, the proper characterization of complex/heterogeneous materials is still an extremely challenging task, since the majority of characterization methods analyze either the average characteristics of the whole material [1] or a narrow area of specific interest [2,3]. On the other hand, the efficient processing/recycling of complex materials/wastes should be strongly supported by their characterization from the viewpoint of economic and environmental concerns [2].

## 2. The Special Issue

The Special Issue contains 10 papers from around the globe on the topics related to the “Characterization and Processing of Complex Materials”, and specifically discusses the following contents.

Wu et al. [4] and Zhou et al. [5] reported their nanobubble studies. Wu et al. [4] developed a method to control the crystalline and morphology of calcium carbonate with the aid of nanobubbles and showed the structure transition from vaterite to calcite. They explained that nanobubbles can coagulate with calcite, work as a binder between calcite and vaterite and accelerate the transformation from vaterite to calcite. They also explained their proposed mechanism by the potential energy calculated using the extended DLVO (Derjaguin–Landau–Verwey–Overbeek) theory. Zhou et al. [5] studied five kinds of nanobubbles (N_2_, O_2_, Ar + 8%H_2_, air and CO_2_) in deionized water and a salt aqueous solution prepared using the hydrodynamic cavitation method. They measured the mean size and zeta potential of the nanobubbles using a light scattering system, while the pH and Eh of the nanobubble suspensions were measured as a function of time. The nanobubble stability was predicted and discussed by the total potential energies between two bubbles by the extended Derjaguin–Landau–Verwey–Overbeek (DLVO) theory.

Fukushima et al. [6,7] reported their studies on the modeling and simulation of electrical disintegration. Fukushima et al. [6] developed a simulation method using equivalent circuits of granite to better understand the crushing process with high-voltage (HV) electrical pulses that have been previously identified as a selective liberation method. Using their simulation works, they calculated the electric field distributions, produced heat and temperature changes in granite when an electrical pulse was applied. Their results suggested that the void volume in each mineral is important in calculating the electrical properties and confirmed that considering both the electrical conductivity and dielectric constant of granite can more accurately represent the electrical properties of granite under HV electric pulse application. Fukushima et al. [7] prepared equivalent circuit models in order to represent the electric and dielectric properties of minerals and voids in a granite rock sample, considering HV electrical pulse application. Using the electric and dielectric properties and the mineral compositions measured, they prepared ten patterns of equivalent circuit models. The values of the electric circuit parameters were determined from the known electric and dielectric parameters of the minerals in granite. The average calculated data of the electric properties of granite agreed with the measured data. 

Elphick et al. [8] applied their scanning electron microscopy (SEM)-based non-destructive imaging technique for the characterization of nanoparticles synthesized using X-ray radiolysis and the sol-gel method. They observed the interfacial conditions between the nanoparticles and the substrates by subtracting images taken by SEM at controlled electron acceleration voltages to allow backscattered electrons to be generated predominantly below and above the interfaces. They found the interfacial adhesion to be dependent on the solution pH used for the particle synthesis or particle suspension preparation, proving the change in the particle formation/deposition processes with pH as anticipated and in agreement with the prediction based on the DLVO theory. 

Kanari et al. [9,10,11,12] reported their characterization results of chromite concentrate [9], copper anode slime [10,12] and alkali ferrates synthesized from industrial ferrous sulfate [11]. Their characterization consists of inductively coupled plasma atomic emission spectroscopy (ICP-AES), SEM-EDS and X-ray diffraction in order to extract information on the chemical composition, morphology and mineralogical composition of their products prepared under different conditions. 

Zhong and Xu [13] reported high-temperature mechanical behaviors of SiO_2_-based ceramic cores for the directional solidification of turbine blades. They conducted isothermal uniaxial compression tests of ceramic core samples on a Gleeble-1500D mechanical simulator with an innovative auxiliary thermal system and investigated the stress–strain results and macro- and micro-structures of SiO_2_-based ceramic cores. They characterized the microstructures using SEM. Based on their experimental data, they established a nonlinear constitutive model for high-temperature compressive damage. The statistical results of the Weibull modulus showed that the stability of hot deformation increases with the increase in temperature. They confirmed the applicability of their model by comparing their results between the predictions of the nonlinear model and the experimental values.
